# Personality Factors Predicting Smartphone Addiction Predisposition: Behavioral Inhibition and Activation Systems, Impulsivity, and Self-Control

**DOI:** 10.1371/journal.pone.0159788

**Published:** 2016-08-17

**Authors:** Yejin Kim, Jo-Eun Jeong, Hyun Cho, Dong-Jin Jung, Minjung Kwak, Mi Jung Rho, Hwanjo Yu, Dai-Jin Kim, In Young Choi

**Affiliations:** 1 Department of Creative IT Engineering, Pohang University of Science and Technology, Pohang, Republic of Korea; 2 Department of Psychiatry, College of Medicine, Seoul St. Mary’s Hospital, The Catholic University of Korea, Seoul, Republic of Korea; 3 Department of Medical Informatics, College of Medicine, The Catholic University of Korea, Seoul, Republic of Korea; Universidad de Granada, SPAIN

## Abstract

The purpose of this study was to identify personality factor-associated predictors of smartphone addiction predisposition (SAP). Participants were 2,573 men and 2,281 women (*n* = 4,854) aged 20–49 years (Mean ± SD: 33.47 ± 7.52); participants completed the following questionnaires: the Korean Smartphone Addiction Proneness Scale (K-SAPS) for adults, the Behavioral Inhibition System/Behavioral Activation System questionnaire (BIS/BAS), the Dickman Dysfunctional Impulsivity Instrument (DDII), and the Brief Self-Control Scale (BSCS). In addition, participants reported their demographic information and smartphone usage pattern (weekday or weekend average usage hours and main use). We analyzed the data in three steps: (1) identifying predictors with logistic regression, (2) deriving causal relationships between SAP and its predictors using a Bayesian belief network (BN), and (3) computing optimal cut-off points for the identified predictors using the Youden index. Identified predictors of SAP were as follows: gender (female), weekend average usage hours, and scores on BAS-Drive, BAS-Reward Responsiveness, DDII, and BSCS. Female gender and scores on BAS-Drive and BSCS directly increased SAP. BAS-Reward Responsiveness and DDII indirectly increased SAP. We found that SAP was defined with maximal sensitivity as follows: weekend average usage hours > 4.45, BAS-Drive > 10.0, BAS-Reward Responsiveness > 13.8, DDII > 4.5, and BSCS > 37.4. This study raises the possibility that personality factors contribute to SAP. And, we calculated cut-off points for key predictors. These findings may assist clinicians screening for SAP using cut-off points, and further the understanding of SA risk factors.

## 1. Introduction

The number of smartphone users is increasing worldwide; about 40 million people (78.6% of the population) in South Korea have smartphones [1]. As smartphones become more popular, concerns about the negative consequences of their overuse are also increasing. Adverse consequences of overuse include physical health-related problems, such as musculoskeletal disorders of the hand, wrist and neck [2, 3], ocular symptoms [4] and elevated risk of psychopathologies such as attention deficit [5], aggression and sleep disturbance [6].

Excessive use of smartphones has generated terms such as “problematic mobile phone use”, “mobile phone addiction”, and “smartphone addiction” (SA). However, currently, there are no established diagnostic criteria for a disorder characterized by excessive patterns of smartphone use, and there is still controversy over whether it is appropriate to apply the word “addiction” to such use. Indeed, to date, the only support for this phenomenon on being an addiction comes from exploratory studies relying on self-reports or clinical case studies [7].

Despite this conceptual limitation, there is a growing need to understand this condition. Through exploratory factor analysis, Lin et al. demonstrated that SA has several similar symptoms to substance-related and addictive disorders from the *Diagnostic and Statistical Manual of Mental Disorders*, *5*^*th*^ *Edition* (DSM-5), including compulsive behavior, tolerance, withdrawal, and functional impairment [8]. Kwon et al. similarly reported that smartphones caused symptoms of addiction similar to the effects of substances, including overuse, tolerance, withdrawal, daily-life disturbance, and positive anticipation [9]. Kuss and Griffiths founded that excessive smartphone use can decrease real-life social interaction, lower academic performance, and negatively affect relationships [10]. In a previous study of 10,191 adolescent smartphone users, 30% of participants showed tolerance, 36% showed withdrawal, 27% showed use that was longer than intended, 18% experienced unsuccessful attempts at reducing, and 10% showed functional impairment of close relationships [11]. In other words, excessive use of smartphone might interfere with important aspects of daily life, as it may lead to disproportionate involvement of smartphone use one’s motivated behaviors in work or interactions with family members and friends. Therefore, the present article will consider excessive smartphone use as a behavior that can be conceptualized within the framework of addiction.

In 2011, the Korean National Information Society Agency developed smartphone addiction proneness scale (K-SAPS) following earlier studies examining Internet and mobile phone addiction and relevant clinical expertise. The complete set of items is divided into four subdomains: tolerance, withdrawal, virtual life orientation, and disturbance of adaptive functions [12]. Results obtained in 2014 using the K-SAPS indicate that 14.2% of South Korean smartphone users aged 10–59 years are in high-risk and at-risk groups for SA. This figure has increased from 11.8% in 2013; smartphones therefore pose potential for addiction risk [1]. Nonetheless, not all people who use smartphones become SA-prone. This implies that SA is affected by pre-existing factors that increase the likelihood that an individual will be affected.

Previous studies indicate that personality traits may affect addiction [13]. Specifically, novelty- or sensation-seeking traits are significantly stronger among drug users than among nonusers [14–16]. In studies of Internet addiction, Ko et al. found that reward dependence was decreased and novelty seeking was increased among Internet-addicted participants [17]; in contrast, Wu et al. reported that addictive tendencies were positively correlated with outcome expectancies and impulsivity, but negatively associated with Internet self-efficacy [18]. In a similar survey, Kuss et al. identified increased neuroticism and low agreeableness as risk factors for Internet addiction [19]. Regarding smartphones, problematic mobile phone use is related to extraversion but not neuroticism [20], although anxiety levels and frequency of neurotic personality traits increase SA severity [21].

The Behavioral Inhibition System/Behavioral Activation System (BIS/BAS) scales were developed to assess personality dimension from Gray’s reinforcement sensitivity theory (RST) [22]. The BIS and BAS are two basic brain systems that control human behavior: the BIS is activated by conditioned stimuli associated with termination of reward or punishment; the BAS is activated by stimuli associated with reward or termination of punishment [23]. So, BIS/BAS personality characteristics may be associated with addiction [24]. The BAS is positively associated with cue-elicited craving in alcoholics [25]; elevated BAS levels correlate with lifetime history of alcohol abuse [26]. College students with Internet addiction have been found to score higher on both BIS and BAS scales [27].

Consistently, individuals who are impulsive and disinhibited are more prone to drug addiction. Impulsivity is a personality factor that has been explained as selecting a smaller reward that may be got immediately over a larger reward that may be obtained after a delay. And selecting the large delayed reward while foregoing the small immediate reward has been explained as a self-control choice [28]. Impulsivity has been related to addictive behavior and diminished control that failure to resist an impulse, drive, or temptation is an essential feature of addiction [29]. Furthermore, self-control has been demonstrated to predict problematic video-game playing in a longitudinal setting [30].

As abovementioned, much research has examined the relationship between personality and addiction; however, few studies have examined personality-based factors affecting SA risk. Knowing what factors distinguish SA from non-SA may help in identifying which individuals may have SA as well as those who might be at an elevated risk of developing it. We therefore aimed to examine personality characteristics (BIS/BAS, impulsivity, and self-control), demographic information, and average smartphone usage hours as risk factors for SA predisposition. In order to identify risk factors, we first identified predictors of SA predisposition, and then derived casual relationships between SA predisposition and its predictors. We also computed optimal cut-off points of the risk factors in order to support practical applications.

## 2. Material and Methods

### 2.1. Participants

Data were collected using an online survey conducted by a professional polling company (Hankook Research, Inc.) from November 26 to December 26, 2014. Participants were 5,003 native Koreans aged 20–49 years from metropolitan areas in South Korea. However, 149 (3%) of them were excluded because they did not own a smartphone. In total, 4,854 participants (2,573 male, 2,281 female) with smartphones completed the entire questionnaire. The mean age for male and female participants was 34.12 years (SD = 7.39 years) and 32.73 years (SD = 7.59 years), respectively. This study was approved by the Institutional Review Board of Seoul St. Mary’s Hospital (IRB number: KC15EISI0103). Informed consent was obtained online from all participants prior to participation; participants who refused to provide consent were excluded.

### 2.2. Materials

The survey was comprised of the following questionnaires: the K-SAPS for adults, BIS/BAS, Dickman Dysfunctional Impulsivity Instrument (DDII), and Brief Self-Control Scale (BSCS). All questionnaires were self-administered. Participants provided their demographic information (age, gender, educational level, and occupational and marital status) and smartphone usage pattern (weekday or weekend average usage hours and main use).

#### 2.2.1. K-SAPS

The K-SAPS was developed by the National Information Society Agency to assess SA (Cronbach’s α = 0.814) [12]. It is composed of 15 items; responses used a 4-point Likert scale (1: *not at all*, 4: *always*). The scale contains four subdomains: (1) disturbance of adaptive functions, (2) virtual life orientation, (3) withdrawal, and (4) tolerance. The sum of all scores or the sums of subdomain scores were used to classify participants into high-risk, at-risk, and normal-user groups. Regarding participants’ classification, T-score of 70 and 65 were used for the high-risk and at-risk groups, respectively. Participants were classified as high-risk if their total score exceeded 44, or if their subdomain scores exceeded 15, 13, and 13 for disturbance of adaptive function, withdrawal, and tolerance, respectively. Participants were classified as at-risk if their total score was 40–43, or if their score for disturbance of adaptive functions exceeded 14. Other participants were classified as normal users. In the present study, participants in the high-risk and at-risk groups were defined as SA predisposition (SAP); other participants were deemed non-SAP. In this study’s sample, Cronbach’s alpha was 0.865, and Spearman’s correlation coefficients between total SAPS score and scores on its four subdomains (disturbance of adaptive functions, virtual life orientation, withdrawal, and tolerance) were 0.804, 0.865, 0.828, and 0.796, respectively.

#### 2.2.2. BIS/BAS

BIS and BAS are general motivation systems that underlie behavior and affect [23, 31]. The BIS responds to cues associated with punishment; the BAS responds to those associated with reward. The BIS and BAS questionnaire scales assess BIS (7 items) and three subdomains of BAS: Drive (4 items), Fun-Seeking (4 items), and Reward Responsiveness (5 items) [22]. Items on the BIS scale assess reactions to anticipated punishment. Items in the BAS-Drive subscale assess persistent pursuit of desired appetitive goals. Items in the BAS Fun-Seeking subscale assess desire for new rewards and willingness to spontaneously approach potentially rewarding events. Items in the BAS-Reward Responsiveness subscale assess positive responses to actual or anticipated rewards. Responses used a 4-point scale (1: *strongly disagree*, 4: *strongly agree*). Cronbach’s alpha was 0.76 for the BIS, 0.83 for the BAS, 0.80 for BAS-Drive, 0.70 for BAS Fun-Seeking, and 0.65 for BAS-Reward Responsiveness [32]. In this study’s sample, Cronbach’s alpha was 0.869, 0.787, 0.781, and 0.772 for BIS, BAS-Drive, BAS-Fun Seeking, and BAS-Reward Responsiveness, respectively.

#### 2.2.3. DDII

The DDII measures impulsivity [33]. Twelve of 23-items in the DDII were selected to evaluate dysfunctional impulsivity. The DDII’s reliability and validity have been supported [33]. The Cronbach’s alpha was 0.74 and 0.85 for functional impulsivity and dysfunctional impulsivity, respectively [33]. Dysfunctional impulsivity is the tendency to act with less forethought, and this tendency causes inaccurate performance [34]. The DDII allows responses of true (1) or false (0); total scores range from zero to 12. Higher scores indicate greater dysfunctional impulsivity. In this study’s sample, Cronbach’s alpha was 0.769.

#### 2.2.4. BSCS

The BSCS is a 13-item measure of self-control; it focuses on processes that involve self-control directly, rather than distal self-control behavioral outcomes [35]. Responses to items use a 5-point scale from 1 (strongly disagree) to 5 (strongly agree); higher scores indicate lower self-control. The scale is highly internally consistent (α = 0.85) [35]. In this study’s sample, Cronbach’s alpha was 0.725.

### 2.3. Statistical Analysis

Data analysis used three steps: (1) identifying predictors using logistic regression, (2) deriving causal relationships between SAP and its predictors using Bayesian belief networks (BN), and (3) computing optimal cut-off points for SAP predictors using the Youden index.

#### 2.3.1. Logistic regression

We used *t*-tests to compare demographic information and personality traits between the SAP and non-SAP groups. To identify SAP predictors, we assessed the effect of demographic information and personality traits to SAP and non-SAP using multivariate logistic regression. Variables with *p*-values<0.05 were considered predictive; 95% confidence intervals were used.

#### 2.3.2. BN

We used the BN to identify risk factors among the predictors that directly related to SAP. The BN is a statistical graphical model that represents probabilistic conditional relationships between random variables [36]. The BN’s structure consists of nodes (random variables) and directed edges between nodes that indicate probabilistic conditional dependency. A node of interest (SAP) is described by the set of other nodes that direct the node of interest. The BN structure describes the data well when the joint probability of the nodes is maximized. As joint probability varies depending on the direction of edges, causes and results may be inferred between the node of interest and directing nodes.

To estimate the BN structure, we first discretized predictors into 0%-25%, 25%-50%, 50%-75%, and 75%-100% categories. We derived the initial BN by adding edges between SAP and the top three predictors of logistic regression whose odd ratio was larger or smaller than 1 with *p*-values < 0.05. We then used Bayesian Information Criterion scores to iteratively add edges on the BN with the largest joint probability and the small number of edges [37]. We used the *bnlearn* R package with hill-climbing learning [38].

#### 2.3.3. Optimal cut-off on ROC curve

For each continuous variable predictor, we found an optimal cut-off point that predicted SAP with the highest sensitivity and specificity. The cut-off predictors provide an immediate intuition on risk of SAP. Using the cut-off, each predictor may be characterized as a dichotomous variable with a value greater-than, less-than, or equal to the cut-off.

The optimal cut-off point maximizes sensitivity + specificity on the receiver operating characteristic (ROC) curve in logistic regression [39, 40]. Logistic regression classifies data into dichotomous outcomes (true or false) based on a specific threshold y*. Because sensitivity and specificity are trade-off depending on the value of y*, we need to choose a ‘good’ y* (0 < y* < 1) that ensures both high sensitivity and specificity. The optimal y* that maximizes both sensitivity and specificity is the point on ROC curve that has maximum vertical distance between the ROC curve and the diagonal line, which is known as Youden index (= sensitivity + specificity -1) [39, 41–43]. Detailed derivation of the optimal cut-off can be found in S1 Appendix.

We performed this analysis on each continuous predictor variable. We first created equally sized samples of the SAP and non-SAP, and then conducted logistic regression of each predictor. We then computed the optimal cut-off points. We repeated this process ten times to calculate the average optimal cut-off. We used the R 3.1.1 with the *pROC* package to calculate the optimal cut-off [44].

## 3. Results

Table 1 provides participants’ demographic characteristics. About 13.4% (N = 652) of participants were in the SAP group (9.4% of all males; 17.9% of all females). Mean values of variables differed significantly between the SAP and non-SAP groups (Table 2). Mean K-SAPS scores were 43.0 and 29.6 in the SAP and non-SAP groups, respectively. SAP group members scored higher on the BIS/BAS, dysfunctional impulsivity, and low self-control, and indicated longer mean weekday and weekend usage hours.

**Table 1:** Demographic characteristics of participants (N = 4,854).

Variables		SAP N (%)	Non-SAP N (%)	χ^2^ (*p* value)
**Gender**				74.89 (< .001)
	Men	243 (9.4)	2330 (90.6)	
	Women	409 (17.9)	1872 (82.1)	
**Age**				30.71(< .001)
	20–29	258 (16.0)	1353 (84.0)	
	30–39	297 (13.9)	1836 (86.1)	
	40–49	97 (8.7)	1013 (91.3)	
**Educational level**				0.001 (.969)
	High school graduate or lower	182 (13.4)	1776 (86.6)	
	College graduate or higher	470 (13.4)	3026 (86.6)	
**Occupational status**				16.2 (.003)
	Employee	339 (52.0)	2361 (56.2)	
	Professional	71 (10.9)	468 (11.1)	
	Student	117 (17.9)	556 (13.2)	
	No occupation	99 (15.2)	564 (13.4)	
	Others	26 (4.0)	253 (6.1)	
**Marital status**				5.24 (.073)
	Single	340 (14.5)	1997 (85.5)	
	Married	294 (12.3)	2097 (87.7)	
	Others	18 (14.3)	108 (85.7)	

**Table 2:** Means and standard deviations of the variables between SAP and non-SAP group.

Variables	SAP Mean (SD)	Non-SAP Mean (SD)	T	Cohen’s d (effect-size r)
**K-SAPS**	43.0 (3.1)	29.6 (6.0)	-87.57***	2.81 (0.81)
**BIS**	20.7 (2.8)	18.6 (3.2)	-17.22***	0.70 (0.33)
**BAS-Drive**	10.6 (1.9)	9.1 (2.0)	-17.64***	0.77 (0.36)
**BAS Fun-Seeking**	11.1 (1.8)	9.6 (2.1)	-19.39***	0.77 (0.36)
**BAS-Reward Responsiveness**	15.0 (2.0)	12.9 (2.8)	-23.12***	0.86 (0.40)
**DDII**	5.3 (3.0)	3.5 (2.7)	-14.28***	0.63 (0.30)
**BSCS**	40.8 (6.1)	35.0 (6.2)	-22.57***	0.94 (0.43)
**Weekday average usage hours**	6.3 (4.4)	4.2 (3.6)	-11.46***	0.52 (0.25)
**Weekend average usage hours**	6.9 (4.6)	4.2 (3.8)	-13.73***	0.64 (0.30)

*Note*: SD (95%standard deviation)

***p < .001.

Regarding smartphone usage, this study’s participants used smartphones primarily for web surfing (36.8% in total, 40.1% among men, 33.1% among women), followed by messenger services (29.1% in total, 25.3% among men, 33.5% among women), games (12.5% in total, 14.2% among men, 10.5% among women), social network services such as blogs, Facebook, and Twitter (8.9% in total, 8.5% among men, 9.4% among women), and entertainment such as listening music or watching movies (6.0% in total, 6.2% among men, 5.7% among women) except for phone calls and short message services (SMS). Males in both groups and females in the non-SAP group used smartphones mainly for web surfing; however, females in the SAP group mainly used smartphones for applications with messenger functions, such as Kakao Talk and Line, which are very popular in South Korea (results not shown).

Logistic regression analysis identified six predictors: gender (female), weekend average usage hours, and scores on BAS-Drive, BAS-Reward Responsiveness, DDII, and BSCS (Table 3). Women were 1.46 times more likely to be addicted to smartphones than men. A one-hour increase in weekend average usage caused the probability of SAP to increase 1.08 times. Higher scores on BAS-Drive, BAS-Reward Responsiveness, DDII, and BSCS also increased the probability of SAP by 1.10, 1.02, 1.09, and 1.13 times, respectively. The area under the ROC curve (AUC) was 0.8279 with a 95% confidence interval of ±0.0187.

**Table 3:** Odds ratio and *p*-value for predictors in logistic regression.

Variables		Odd ratio, exp(beta)	Estimate (beta)	Estimate's 95% CI	p-value
**Gender (women)**		1.462	0.380	0.022	.001**
**Age**					
	30s	1.136	0.128	0.020	.266
	40s	0.848	-0.165	0.014	.067
**Education level (college graduate or higher**		1.041	0.040	0.021	.730
**Occupation**					
	Professional	0.888	-0.118	0.017	.498
	Student	0.911	-0.094	0.028	.601
	Unemployed	0.879	-0.129	0.090	.703
	Others	1.131	0.123	0.038	.589
**Marital status**					
	Married	0.975	-0.025	0.022	.771
	others	1.033	0.032	0.046	.831
**Weekday average usage hours**		1.013	0.013	0.005	.600
**Weekend average usage hours**		1.079	0.076	0.004	.001**
**BIS**		0.977	-0.023	0.004	.320
**BAS**					
	Drive	1.105	0.267	0.008	.000**
	Fun-Seeking	1.306	0.021	0.006	.595
	Reward-Responsiveness	1.021	0.100	0.008	.009*
**DDII**		1.088	0.084	0.005	.000**
**BSCS**		1.134	0.125	0.002	.000**

*Note*: SD (95% standard deviation)

**p < .005

* *p* < .01, Age are compared with 20s, occupations are compared with employee, and marital status are compared with single.

**Fig 1:**
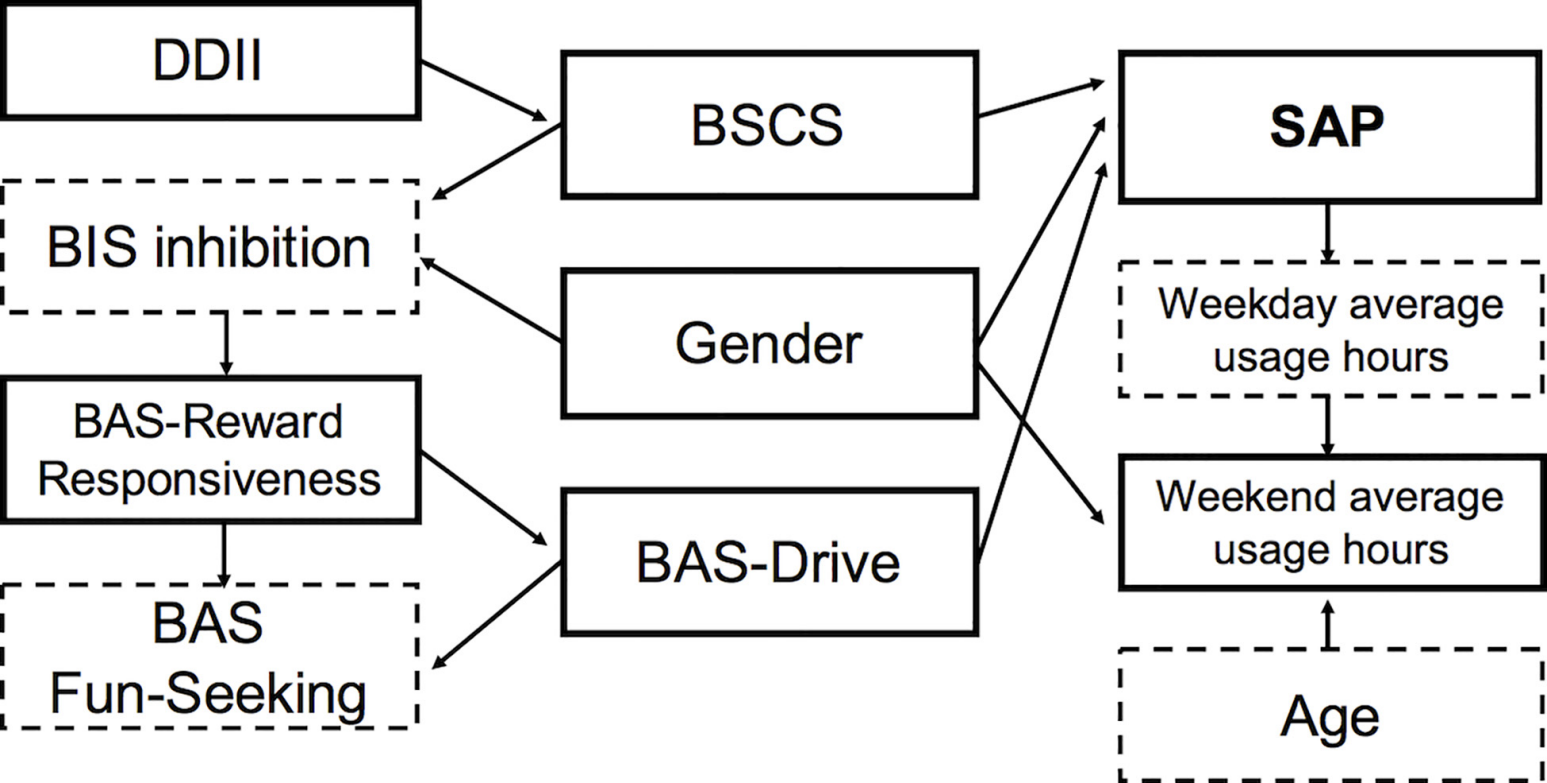
Bayesian belief network for SAP and variables. Arrows are directed from cause to result. Solid boxes = Variables with *p*-value < 0.01, Dashed boxes = Variables with *p*-value ≥ 0.01 in logistic regression.

We derived the probabilistic conditional relationship between SAP and its predictors (Fig 1), and found that gender, BAS-Drive and BSCS scores strongly affected SAP. SAP consequently affected longer average weekday usage hours, and it affected longer average weekend usage hours. Other predictors such as BAS-Reward Responsiveness and DDII indirectly affected SAP via BAS-Drive or BSCS. Age did not directly affect SAP; however, it affected weekend usage hours, which are related to SAP. We thus found that the six predictors consist of three direct causes (gender, BAS-Drive and BSCS), two indirect causes (BAS-Reward Responsiveness and DDII), and one result (weekend average usage hours).

To examine the exact probabilities of SAP’s direct causes, we examined the conditional probabilistic relationship between BAS-Drive and BSCS (Table 4). Participants with BAS-Drive scores of 11–16 and high BSCS scores of 37–40 or 40–65 had higher probability of SAP than participants with low BAS-Drive or BSCS scores. The probabilities of SAP given BAS-Drive scores over 11 and BSCS scores over 37 were 0.455 to 0.475, respectively, which are considerably higher than the priori probability regardless of BAS-Drive and BSCS scores, 0.122 (i.e.,592/4854, 592 SAP participants out of 4854).

**Table 4:** Conditional probabilities of SAP given BAS-Drive and BSCS scores.

Gender	BAS- Drive	BSCS	Non- SAP	SAP	Gender	BAS- Drive	BSCS	Non- SAP	SAP
M	4–8	13–32	0.99	0.01	F	4–8	13–32	0.97	0.03
M	8–9	13–32	1.00	0.00	F	8–9	13–32	0.98	0.02
M	9–11	13–32	0.97	0.03	F	9–11	13–32	0.98	0.02
M	11–16	13–32	0.97	0.03	F	11–16	13–32	0.84	0.16
M	4–8	32–37	0.98	0.02	F	4–8	32–37	0.95	0.05
M	8–9	32–37	0.93	0.07	F	8–9	32–37	0.92	0.08
M	9–11	32–37	0.96	0.04	F	9–11	32–37	0.92	0.08
M	11–16	32–37	0.91	0.09	F	11–16	32–37	0.73	0.27
M	4–8	37–40	0.96	0.04	F	4–8	37–40	0.95	0.05
M	8–9	37–40	0.89	0.11	F	8–9	37–40	0.84	0.16
M	9–11	37–40	0.85	0.15	F	9–11	37–40	0.78	0.22
M	**11–16**	**37–40**	**0.54**	**0.46**	F	**11–16**	**37–40**	**0.56**	**0.44**
M	4–8	40–65	0.94	0.06	F	4–8	40–65	0.78	0.22
M	8–9	40–65	0.86	0.14	F	8–9	40–65	0.80	0.20
M	9–11	40–65	0.79	0.21	F	9–11	40–65	0.66	0.34
M	**11–16**	**40–65**	**0.51**	**0.49**	F	**11–16**	**40–65**	**0.51**	**0.49**

*Note*: Probability of SAP when BAS-Drive score is over 11 and BSCS score is over 37 is 0.455 to 0.475. Probability of SAP of females tends to be higher than probability of males as BAS-Drive and BSCS score increase. M = Male, F = Female.

**Fig 2:**
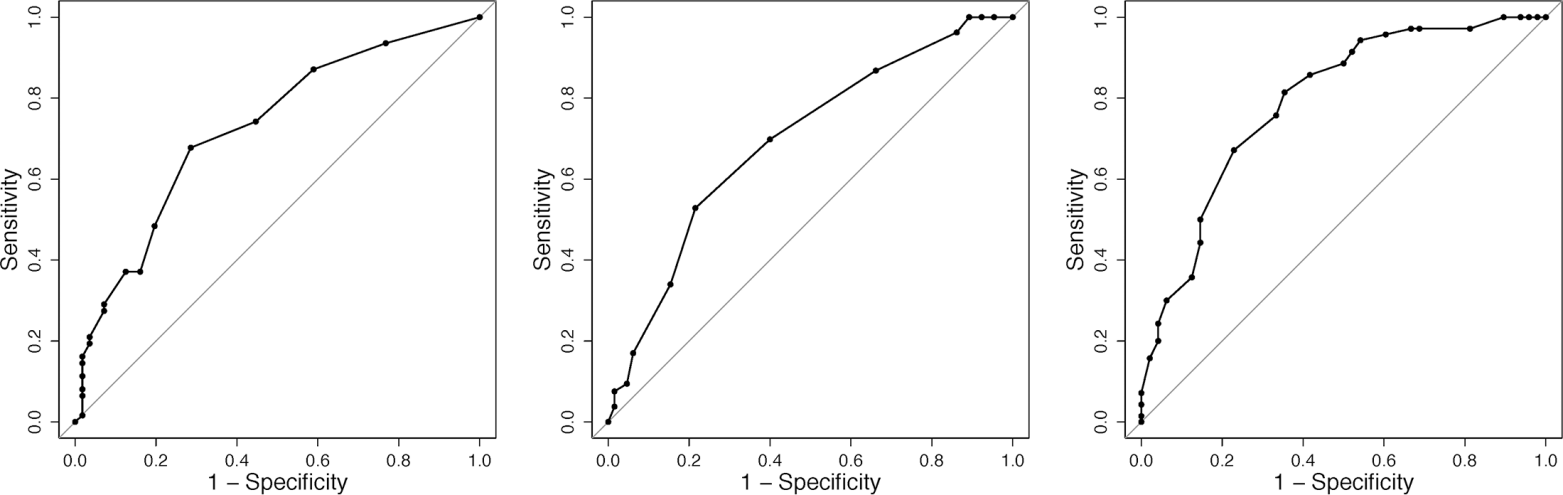
ROC curve for logistic regression. (a) average weekend usage hours, AUC = 0.69 ± 0.02, (b) BAS-Drive, AUC = 0.72 ± 0.03, (c) BSCS, AUC = 0.76 ± 0.02. Each point on the ROC curve represents a cut-off point for binary classification (Table 5).

To compute the optimal cut-offs for the predictors using logistic regression, we first derived the ROC curve for logistic regression with each predictor (Fig 2). We found the optimal cut-off that maximized the sensitivity and specificity for each predictor (Table 5). As the cut-off point increased, sensitivity increased and specificity decreased. For example, sensitivity and specificity were maximized when weekend average usage hours were > 4.5, yielding a prediction of SAP for this range of values (i.e., weekend average usage hours of < 4.5 would yield a prediction of non-SAP). We repeated this process ten times to compute the average optimal cut-off points (Table 6). It is notable that the cut-offs for BAS-Drive (> 10.0) and BSCS (> 37.4) are consistent with the BN’s cut-off points at which the probability of SAP was maximized (BAS-Drive > 11 and BSCS > 37).

**Table 5:** Cut-off points with sensitivity and specificity.

Weekend average usage hours	Cut-off point [hours]	1.5	2.5	3.5	4.5*	5.5	6.5	7.5	9.0	10.5	11.5
	Sensitivity [%]	95.4	83.1	72.3	**64.6**	46.2	38.5	35.4	24.6	21.5	20.0
	Specificity [%]	26.4	50.9	64.2	**73.6**	83.0	88.7	88.7	92.5	92.5	92.5
BAS-Drive	Cut-off point	4.5	6.0	7.5	8.5	9.5	10.5*	11.5	12.5	13.5	15.0
	Sensitivity	100.0	100.0	98.6	94.2	78.3	**52.2**	36.2	17.4	7.3	2.9
	Specificity	4.1	6.1	8.2	32.7	55.1	**83.7**	89.8	95.9	98.0	100.0
BSCS	Cut-off point	32.5	33.5	34.5	35.5	36.5	37.5*	38.5	39.5	40.5	41.5
	Sensitivity	96.4	92.7	89.1	89.1	87.3	**85.5**	78.2	65.5	52.73	45.45
	Specificity	25.4	39.7	41.3	42.9	47.6	**54.0**	60.3	68.3	74.6	80.95

Note

* Optimal cut-off

**Table 6:** Average cut-off points for the five predictive risk factors.

	Averaged optimal cut-off point	SD
Weekend average usage hours	4.45	1.1
BAS-Drive	10.0	0.6
BAS-Reward Responsiveness	13.8	0.4
DDII	4.5	0.8
BSCS	37.4	1.0

*Note*: SD (95% standard deviation)

## 4. Discussion

The primary objective of this study was to identify SAP risk factors. SAP predictors were female gender, weekend average usage hours, personality traits of pursuit for desired appetitive goals, positive responses to anticipated rewards, dysfunctional impulsivity, and low self-control.

In the present study, women were 1.46 times more prone to addiction to smartphones than men. Consistently, previous studies suggested that females are more addicted to smartphones [21] and females are also likely to experience more frequent problematic use of mobile phones [45]. However, the reason for observed gender-related differences in SAP is not well understood. Differences in usage contents, motivation for smartphone use and features of smartphone such as ease of use may contribute to the findings. Characteristics of contents and motivations can affect behavior and may serve as risk factors for developing behavioral addiction [46, 47]. Alexander et al. [48] reported that people who extensively use smartphones for social purposes develop smartphone habits faster, which in turn might lead to addictive smartphone behavior and men use their smartphones less for social purposes. Chen [49] identified that women place greater importance on using smartphones to socialize than men do, and interest-oriented use is more salient among males than among females. In the present study, women in the SAP group mainly used smartphones for messenger services, whereas men in the same group mainly used smartphones for web surfing. The difference in proportions of usage may also partly explain why being female had an effect on smartphone use. Nonetheless, further research is needed to examine gender-related predictors of SAP and which contents and motivations for smartphone use might relate to SAP.

In this study, participants in the SAP group showed longer mean weekday and weekend usage hours. This is consistent with a Swiss study of 1,519 young people, wherein a longer duration of smartphone use on a typical day was a significant predictor of SA [50]. Additionally, the present study showed that SAP group members used smartphones longer over the weekend than on weekdays, and only weekend average usage hours predicted SAP. This may be because three quarters of SAP group members worked on weekdays.

We found that BIS and BAS scores were both higher in the SAP group than in the non-SAP group; however, only BAS predicted SAP in the logistic regression analysis, excepting BAS-Fun Seeking. Consistent with our findings, BAS is an important predictor whereas BIS has reverse effects or is not a significant predictor on addictive and risky behavior [51]. Additionally, individuals with strong BAS are sensitive to reinforcement and motivated to approach rewards [52, 53] and strong BAS elevates the risk of problematic drinking among college students [51, 54] and problematic computer-related behavior among middle school students [55].

As mentioned previously, impulsivity and low self-control are core features of addiction [28, 29]. High impulsivity has been identified as risk factors for addiction to social networking sites among smartphone users [18]. Earlier studies have found that self-control is negatively correlated with alcohol use, illegal drug use, risky sexual behavior, minor illegal behavior, and SA [35, 56]. These findings are corroborated by the results of our study. This means SAP is similar to other behavioral addictions in that regulation of impulsivity and improvement of self-control are important for treatment [57].

Several previous studies have demonstrated a process in which BAS is the basis for impulsivity, which, in turn, provides a tendency toward risky behaviors [58]. On the other hand, some researchers have considered that impulsivity has also been viewed as resulting from an initiation of incentive-motivated, goal-directed behavior [59]. In other words, human behavior can be explained by reciprocal causation of personality factors. As such, we examined SAP’s correlation with BIS, BAS, impulsivity, and self-control, and identified six predictors consisting of three direct causes (female gender, BAS-Drive, and low self-control), two indirect causes (BAS-Reward Responsiveness and dysfunctional impulsivity), and one result (weekend average usage hours). This result suggests that individuals with greater reward dependency and high impulsivity are more prone to engaging in smartphone approach behavior. Additionally, among such individuals, people with a strong desire to pursue the smartphone use and decreased ability to delay their actions tend to initiate smartphone use, and subsequently show an increased frequency, intensity, and duration of such use. Smartphones’ wide availability and easy accessibility may magnify the effects of BAS-Drive and low self-control on SAP [7, 18].

Additionally, we computed the optimal cut-off points that divided participants into the SAP and non-SAP groups, and found preliminary decision rules, as follows: for highest specificity and sensitivity in classifying participants into the SAP group, weekend average usage hours > 4.45, BAS-Drive scores > 10.0, BAS-Reward Responsiveness scores > 13.8, DDII scores > 4.5, and BSCS scores > 37.4.

To our knowledge, this research is the first to investigate these personality factors’ cut-off points for SAP, although earlier research has identified cut-off values for predictors using ROC curves in other areas (e.g., detecting thyroid nodules and acute kidney injury) [60–62]. In practice, dichotomous variables with cut-offs are more useful than continuous ones, as dichotomous variables provide an immediate intuition on risk of SAP. Additionally, cut-off points are easily applied in decision-making regarding people who may be addicted to smartphones. For example, although the continuous BSCS value itself predicts SAP more accurately, an immediate judgment on SAP can be made based on whether BSCS > 37.4. This cut-off may also be used as a SAP self-screening tool, allowing individuals to easily assess their usage hours or personality traits.

Meanwhile, the cut-offs must be interpreted with caution, as decisions made based on optimal cut-off points inevitably include low sensitivity or specificity due to the simplified rule. The sensitivity of BAS-drive > 10.5 and the specificity of BSCS > 37.5 were low (Table 5). Dichotomous classification using cut-offs should be used to inform preliminary and prompt decision rules to help detect SAP, and in combination with more accurate classification methods.

This study has the following limitations. First, the use of the term SA remains highly controversial even though we used the term SAP. Excessive smartphone use can cause maladaptive behavioral difficulties seen in other behavioral addictions, therefore it requires acknowledgment and attention. However, because of the limited findings, a cautious approach should be taken whether or not smartphone overuse should be grouped together with addiction. Second, we used psychometric tool created ad hoc such as K-SAPS to detect SAP. These screening tools are limited to inform as an early detection and only clinical studies are proper to uphold that a certain behavior is pathological [63]. Third, this study’s participants were recruited from a specific region and age range, and were not randomized. In particular, adolescent smartphone usage may reflect a wider range of reasons and behavioral patterns than adult smartphone usage. Fourth, assessment of SAP and the measurement of personality factors relied on self-administered questionnaires. Fifth, relatively few personality factors were measured cross-sectionally, and other psychological factors were not measured. Some researchers have found that people with low self-esteem tended to exhibit problematic mobile phone use, and that people with excessive mobile phone use experienced more depressive symptoms, greater difficulty in expressing emotion, and greater interpersonal anxiety [20, 64]. Loneliness, alexithymia, and obsessive-compulsive behavior have also been linked to behavioral problems such as addictive gambling and Internet addiction [65, 66]. Sixth, this study’s participants were not recruited from a clinical setting; hence, comparison of results from clinical populations with those of this study is required.

Despite these limitations, this study has a notable strength in its large sample size. And this study raises the possibility that personality factors contribute to SAP. That is, individual differences in personality may predict risk and subsequent onset of SA. Further, we calculated cut-off points for key predictors, which may assist clinicians in screening for SAP. Finally, given the paucity of research on SA, this study provides knowledge that will help clinicians’ understanding of the characteristics of smartphone users.

## Supporting Information

S1 Appendix Derivation of optimal cut-off on ROC curve. (DOCX)
