# Supplementary material for: Personality Factors Predicting Smartphone Addiction Predisposition: Behavioral Inhibition and Activation Systems, Impulsivity, and Self-Control
Source: PLoS One. 2016 Aug 17;11(8):e0159788. doi: 10.1371/journal.pone.0159788 (PMC4988723; doi:10.1371/journal.pone.0159788)
Supplement: S1 Appendix — (DOCX) [file pone.0159788.s001.docx]

**S1 Appendix. Derivation of optimal cut-off on ROC curve.**

The optimal cut-off point maximizes sensitivity + specificity on the receiver operating characteristic (ROC) curve in logistic regression (38, 39). Logistic regression classifies data into dichotomous outcomes (true or false). If the value of the logistic function is close to 1, the data is classified as true; if the value is close to 0, then the data is classified as false. More specifically, if the value of the logistic function $1/{(1+e^{-\left( \beta_{0}+\beta x \right)})}$ is greater than a specific threshold $y^{*}$, then the data $x$ is classified as true; if $1/{(1+e^{-\left( \beta_{0}+\beta x \right)})}$ is less than the threshold $y^{*}$, then $x$ is classified as false. In other words,

$$outcome=\left\{ \begin{aligned} true, &if 1/{(1+e^{-\left( \beta_{0}+\beta x \right)})}\geq y^{*} \\ false, &if 1/{(1+e^{-\left( \beta_{0}+\beta x \right)}})< y^{*}. \end{aligned} \right.$$

If $y^{*}$ is close to 0, sensitivity increases to 1 as all data is classified as true; however, specificity simultaneously decreases to 0 as no false data is classified as false. Similarly, if $y^{*}$ is close to 1, sensitivity decreases to 0 as no true data is classified as true; however, specificity simultaneously increases to 1 as all data is classified as false. Consequently, we need a ‘good’ $y^{*}$ (${0<y}^{*}<1$) that ensures both high sensitivity and specificity.

To find the $y^{*}$ that maximizes both sensitivity and specificity, we plot sensitivity and 1 minus specificity at every threshold $y^{*}$; this is known as the ROC curve. After plotting the ROC curve, we can easily detect which $y^{*}$ maximizes sensitivity + specificity. The point that has maximum vertical distance between the ROC curve and the diagonal line is the optimal $y^{*}$ that has largest sensitivity + specificity, which is known as Youden index (= sensitivity + specificity -1) (38, 40-42).

After finding the optimal $y^{*}$, and because $y^{*}$ is the threshold of the logistic function, we transformed the threshold of the logistic function $y^{*}$ into a threshold of data, $x^{*}$.

$1/{(1+e^{-\left( \beta_{0}+\beta x^{*} \right)})}= y^{*}$,

$x^{*}={(\ln\frac{1}{y^{*}}-1+\beta_{0})}/\beta$.
